# Functional Divergence and Toxin Coupling of Cyanobacterial Blooms Across the Lake–River Continuum: Insights from the Lake Taihu Watershed

**DOI:** 10.3390/toxins18020089

**Published:** 2026-02-09

**Authors:** Xiang Wan, Yucong Li, Qingju Xue, Guoxiang Wang, Liqiang Xie

**Affiliations:** 1 College of Environment and Ecology, Jiangsu Open University, Nanjing 210017, China; wanxiang@jsou.edu.cn; 2 State Key Laboratory of Lake and Watershed Science for Water Security, Nanjing Institute of Geography and Limnology, Chinese Academy of Sciences, 73 East Beijing Road, Nanjing 210008, China; 3 School of Environment, Nanjing Normal University, Nanjing 210042, China; 4 School of Ecology and Environment, Anhui Normal University, Wuhu 241002, China

**Keywords:** microcystins, phytoplankton community, *Microcystis*, *Dolichospermum*, lake–river network, hydrodynamic connectivity

## Abstract

While harmful cyanobacterial blooms (HCBs) are extensively characterized in eutrophic lakes, the ecological dynamics of connected river networks remain oversimplified, obscuring the mechanisms of community assembly and toxin distribution across the lake–river interface. This study investigated the spatial heterogeneity of HCBs and microcystins (MCs) in the Lake Taihu watershed, revealing a complex functional divergence between lotic and lentic ecosystems. The rivers functioned as primary nutrient sources, with Total Nitrogen (3.35 ± 1.52 mg·L^−1^) and Total Phosphorus (0.21 ± 0.22 mg·L^−1^) concentrations being 1.7-fold and 1.8-fold higher, respectively, than those in the lake during peak periods. Conversely, the lake acted as a biological sink, supporting a peak cyanobacterial density (3.32 × 10^7^ cells·L^−1^) nearly 1.5 times that of the river network. Phytoplankton community analysis revealed distinct ecological niches: while the lentic lake environment was almost exclusively dominated by colonial *Microcystis* (>90% relative abundance), the lotic river networks harbored a diverse assemblage with significant contributions from filamentous *Oscillatoria* and *Dolichospermum*. Correspondingly, intracellular MC (IMC) in the lake (up to 14.5 μg·L^−1^) significantly exceeded riverine levels (generally <1.0 μg·L^−1^). Despite these compositional differences, toxin dynamics exhibited strong bidirectional coupling (r > 0.75, *p* < 0.01), suggesting a spillover effect where the lake determines the watershed’s toxin burden during rivers outflow period. Redundancy Analysis (RDA) further elucidated that limnetic blooms were primarily regulated by water temperature and pH, whereas riverine communities were strictly constrained by hydrodynamic flow. Consequently, the health risk assessment revealed a highly heterogeneous landscape where, beyond the northern lake bays, specific semi-lentic river segments emerged as cryptic hotspots. These findings demonstrate that while nutrients fuel the system, hydrodynamic conditions act as the ultimate ecological filter determining the spatiotemporal distribution of cyanobacterial risks, necessitating an integrated approach to monitoring the entire lake–river continuum.

## 1. Introduction

The proliferation of harmful cyanobacterial blooms (HCBs) in freshwater ecosystems has escalated into a global environmental crisis, driven by the synergistic effects of anthropogenic eutrophication and climate change [1,2]. Rising temperatures and altered precipitation patterns are increasingly favoring cyanobacterial dominance over eukaryotic phytoplankton [3]. These blooms not only disrupt aquatic food webs and deplete dissolved oxygen but also produce a variety of potent secondary metabolites, among which microcystins (MCs) are the most ubiquitous and concerning. MCs are cyclic heptapeptides characterized by high structural diversity, with over 270 congeners identified to date, differing primarily in their variable amino acids [4]. Among these, MC-LR (Leucine-Arginine), MC-RR (Arginine-Arginine), and MC-YR (Tyrosine-Arginine) are the most frequently detected variants, with MC-LR being particularly notorious for its high chemical stability and potent hepatotoxicity [5]. These toxins pose severe health risks to humans and animals through drinking water contamination, recreational exposure, and bioaccumulation in the food chain [6]. Consequently, the World Health Organization (WHO) has established strict guideline values for MC-LR in drinking water, using it as the standard reference for toxicity assessment [7]. While the urgency of this issue has catalyzed decades of intensive research, the vast majority of studies have historically focused on lentic (standing water) ecosystems, such as lakes and reservoirs, where hydrological stability is intrinsically favorable for cyanobacterial dominance [8].

In contrast, the dynamics of HCBs and MCs in flowing water ecosystems, particularly in river networks connected to eutrophic lakes, remain disproportionately understudied. Historically, rivers have been viewed primarily as simple transport channels that carry nutrients to downstream lakes rather than as active habitats where phytoplankton can grow [9]. This traditional view assumes that the turbulent, fast-moving water in rivers physically washes out slow-growing cyanobacteria, such as *Microcystis*, before they can accumulate significant biomass [10]. However, this assumption is increasingly being challenged by human modifications and environmental changes. The widespread construction of dams and sluice gates has significantly slowed down river flows and created calm, lake-like conditions that act as “incubators” for intense algal blooms [11,12]. Furthermore, complex hydrological disturbances, including wind-driven currents, backflow events, and extreme weather such as typhoons, can override natural flow patterns. These events facilitate the exchange of toxins between lakes and rivers or resuspend overwintering algae from river sediments [13]. Despite these evolving risks, comparative studies that simultaneously quantify toxicological patterns in both lake and river compartments are rare, leaving the interaction mechanisms and potential hidden risks within these networks poorly resolved [14].

Lake Taihu, the third-largest freshwater lake in China, serves as an ideal model system to investigate these complex interactions. As a large, shallow, hyper-eutrophic lake, Taihu has been plagued by perennial cyanobacterial blooms for decades, most notably causing a severe drinking water crisis in 2007 that affected millions of people [15,16]. The lake is connected to a complex, dense network of over 100 inflow and outflow rivers, forming a unique reticular drainage system that facilitates constant material exchange [17]. The watershed is characterized by a highly dynamic hydrodynamic environment, heavily influenced by the East Asian monsoon and strictly regulated by extensive artificial water control projects, such as the Yangtze-Taihu Water Transfer Project [18]. While the bloom dynamics and nutrient mechanisms within the open lake have been extensively characterized [19,20,21], the associated risks in the surrounding river network are frequently oversimplified. These rivers function as both the nutrient source and the recipient of lake water, yet their specific role as potential reservoirs for toxins remains unclear. Understanding the dynamics of these rivers is critical, as they may act not only as transporters but also as active contributors to regional water security risks.

To address these gaps, this study conducted a comprehensive seasonal investigation of harmful cyanobacteria and MCs across the Lake Taihu watershed, covering both the open lake zones and the primary connecting river networks. The specific objectives of this study were to: (1) compare the spatiotemporal distribution patterns of cyanobacterial communities and MC concentrations between the lake and river ecosystems; (2) identify the distinct environmental drivers, such as flow rate and temperature, that regulate bloom formation in rivers compared to the lake; (3) quantify the bidirectional coupling relationship of toxins at the lake–river interface; and (4) assess the specific human health risks associated with MCs in the river network, particularly focusing on identifying overlooked high-risk hotspots. This research aims to provide a scientific basis for integrated watershed management that accounts for the hydrological connectivity of toxic risks.

## 2. Results and Discussion

### 2.1. Spatiotemporal Variations in Physicochemical Parameters

Water temperature (WT) exhibited distinct seasonal patterns ranging from 6.7 °C (winter) to 29.7 °C (summer), with no statistically significant differences observed between the lake and connected rivers (*p* > 0.05). However, unlike the uniform temperature profile, significant spatial heterogeneity was found in nutrient concentrations and biological indicators, revealing a functional divergence between the lotic and lentic ecosystems (Table 1).

Generally, the connected rivers functioned as major nutrient sources, exhibiting consistently higher nutrient levels than the lake zone. Specifically, during August and November, concentrations of Total Nitrogen (TN), Total Phosphorus (TP), and dissolved nutrients (including NH_4_^+^-N, NO_3_^−^-N, and PO_4_^3−^) were significantly higher in the rivers compared to the lake (*p* < 0.05). For instance, in November, the average TN and TP in rivers reached 3.35 ± 1.52 mg·L^−1^ and 0.21 ± 0.22 mg·L^−1^, respectively, which were markedly higher than those in the lake (1.99 ± 1.28 mg·L^−1^ and 0.12 ± 0.09 mg·L^−1^). This distinct gradient confirms that tributary discharge remains the primary conduit for external nutrient loading (e.g., agricultural runoff and domestic wastewater) into Lake Taihu [22,23], supporting the conclusion of Qin et al. [20] that continuous external inputs remain a critical factor in sustaining the lake’s eutrophic state despite long-term restoration efforts.

Chlorophyll-a (Chl-*a*) and pH showed dynamic spatiotemporal shifts, reflecting the interplay between nutrient availability and hydrodynamic stability. In May, the lake zone was characterized by a severe algal bloom, indicated by a peak Chl-*a* concentration of 102.8 ± 104.1 mg·L^−1^ and a significantly elevated pH of 9.3 ± 0.5, likely driven by intense photosynthetic activity which depletes dissolved CO_2_ and shifts the carbonate equilibrium [24]. The contrasting low Chl-*a* levels in rivers during this period, despite high nutrient availability, support the “Residence Time Hypothesis,” which posits that hydrological flushing in flowing rivers prevents the accumulation of slow-growing colony-forming cyanobacteria like *Microcystis*, whereas the lake’s long retention time acts as an incubator for blooms [25].

Notably, a spatial reversal in biomass was observed in August, where Chl-*a* concentrations in the rivers (89.2 ± 192.8 mg·L^−1^) significantly exceeded those in the lake (30.0 ± 11.9 mg·L^−1^). It is important to highlight that the August sampling campaign coincided with a typhoon event. Consequently, the unexpectedly lower Chl-*a* levels in the lake zone were likely attributable to the dilution effect and strong hydrodynamic disturbances caused by the heavy rainfall. Previous studies on Lake Taihu have demonstrated that typhoons can disrupt bloom stability through strong wind–wave mixing and reduced light penetration, effectively causing a temporary bloom dissipation event in open waters [26,27]. By February, although nutrient levels remained high in rivers, Chl-*a* levels dropped to their annual minimum in both zones, confirming that temperature, rather than nutrients, acts as the limiting factor during winter [21].

### 2.2. Seasonal Dynamics of Phytoplankton and Potentially Toxigenic Cyanobacteria

**Figure 1:**
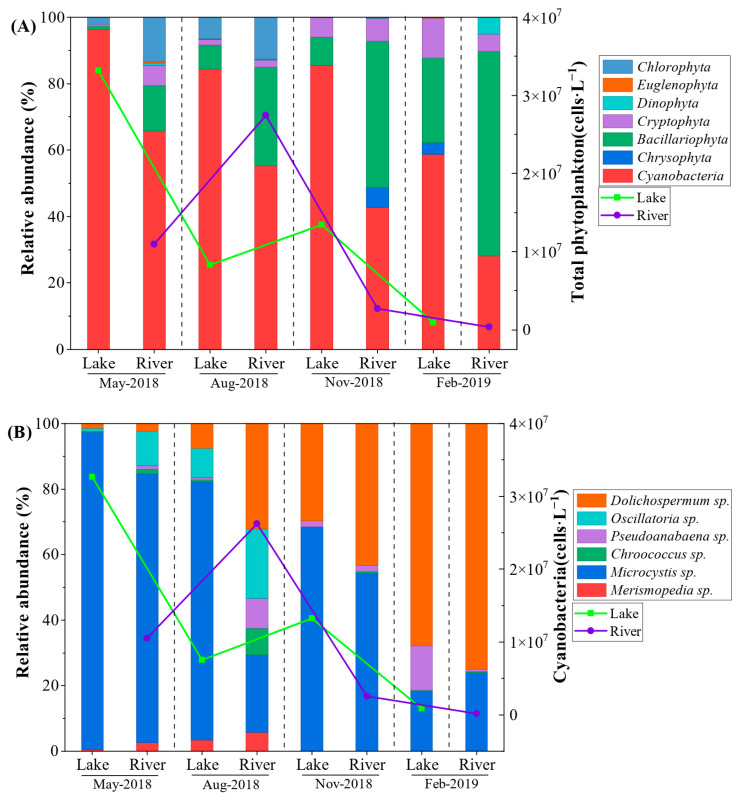
Seasonal dynamics of phytoplankton community structure in Lake Taihu and its connected rivers. (**A**) Relative abundance of phytoplankton at the phylum level. (**B**) Relative abundance of cyanobacteria at the genus level. The stacked bars represent the percentage composition (left *y*-axis), while the line graphs indicate the mean total phytoplankton density (cells·L^−1^, right *y*-axis) across four sampling seasons.

The seasonal succession of phytoplankton communities exhibited marked differences between the lake and river networks (Figure 1A). Cyanobacteria were the omnipresent dominant phylum in the lake zone throughout the study period, accounting for over 80% of the total relative abundance in May, August, and November. This observed dominance of Cyanobacteria in the lake zone during the warm seasons aligns with previous findings by Su et al. [28], who demonstrated that Cyanobacteria consistently prevail in Lake Taihu from late spring through autumn, typically excluding the colder period from January to April. This sustained dominance is largely attributed to the specific morphological traits of bloom-forming cyanobacteria, particularly their intracellular gas vesicles, which provide buoyancy control to maintain position in the euphotic zone of the stratified lake, thereby outcompeting non-buoyant taxa [8]. Similar successional patterns have been documented in other hyper-eutrophic shallow lakes, such as Lake Erie [29], where the stabilization of the water column in summer facilitates the formation of massive cyanobacterial blooms.

Previous studies have demonstrated that diatoms, green algae, and cyanobacteria can all dominate river plankton communities depending on specific hydrological and nutrient conditions [30]. In this study, the connected rivers showed a distinct seasonal shift: while Cyanobacteria dominated during the warm seasons (May and August), the community structure shifted significantly towards *Bacillariophyta* (diatoms) during the colder months (November and February), suggesting a stronger environmental filtering effect in the lotic (riverine) ecosystem compared to the lentic (lake) ecosystem. Specifically, the turbulent flow and lower temperatures in rivers during winter create a physical environment that favors diatoms, which are generally well-adapted to mixed water columns and lower light availability, over the stability-dependent cyanobacteria [30].

Consistent with the Chl-*a* patterns observed in Section 2.1, the total phytoplankton density in the lake peaked in May (3.32 × 10^7^ cells·L^−1^). This observation aligns well with the long-term phenological shifts reported by Deng et al. [21], who demonstrated that warmer spring temperatures have significantly advanced the onset of *Microcystis* blooms, leading to intensified biomass in the spring season. However, in August, the density in the rivers (2.74 × 10^7^ cells·L^−1^) surpassed that of the lake (8.27 × 10^6^ cells·L^−1^), indicating a potential accumulation of biomass in the river network during high-flow conditions. This anomaly challenges the traditional “Residence Time Hypothesis,” which suggests rivers typically wash out biomass [25], indicating that under specific hydrological conditions (e.g., backmixing or extreme runoff), river networks can essentially function as temporary sinks for allochthonous biomass transported from the lake.

Further analysis of the cyanobacterial composition (Figure 1B) highlighted the spatiotemporal dynamics of potentially toxigenic cyanobacteria (PTC), specifically *Microcystis*, *Dolichospermum*, and *Oscillatoria* [4]. *Microcystis* was the omnipresent dominant genus, particularly in the lake zone, where it maintained absolute dominance (>90%) during the bloom outbreak in May. However, significant spatial heterogeneity emerged in August. While the lake zone remained dominated by *Microcystis*, the river network exhibited a shift in community composition, characterized by a substantial increase in filamentous cyanobacteria, including *Oscillatoria* and *Dolichospermum*.

This distinct pattern in the rivers coincided with the prevailing monsoon season, characterized by consistently high wind speeds and punctuated by the typhoon event, suggesting that hydrodynamic disturbances likely favored the resuspension or transport of these filamentous genera over the colonial *Microcystis* [26]. Unlike colonial *Microcystis,* which requires water column stability to form surface scums, filamentous genera such as *Oscillatoria* are often tychoplanktonic (derived from the benthos) or periphytic. Intense hydraulic shear and sediment disturbance caused by typhoons can detach these filaments from the riverbed, elevating their abundance in the water column [26,27]. This hydrodynamic sorting mechanism explains why the lotic environment exhibited a higher diversity of PTCs compared to the lentic zone during the disturbance event.

By winter (February), a seasonal succession was evident as *Dolichospermum* replaced *Microcystis* as the dominant PTC, particularly in the river network, likely reflecting its superior adaptation to lower temperatures compared to *Microcystis*. Additionally, *Dolichospermum* (formerly *Anabaena*) possesses the ability to form akinetes (resting spores) and fix atmospheric nitrogen, physiological traits that provide a competitive advantage under the low-temperature and potentially nitrogen-limited conditions often observed in winter transition periods [8].

### 2.3. Seasonal Dynamics of Microcystins

MCs in the study area were predominantly present in the intracellular form (IMC), with concentrations ranging from undetectable to a maximum of 14.5 μg·L^−1^, whereas extracellular microcystins (EMC) remained consistently low (generally <0.4 μg·L^−1^). This predominance of intracellular microcystins (IMC) aligns with previous observations in Lake Taihu by Su et al. [28] and Zhang et al. [31], where IMC concentrations frequently exceeded 10 μg·L^−1^ while extracellular microcystins (EMC) remained at relatively low average levels (approximately 0.12 μg·L^−1^). Spatially, the lake zone acted as the primary reservoir for intracellular toxins during most of the year. Specifically, in May, November, and February, T−IMC concentrations in the lake were significantly higher than those in the connected rivers (*p* < 0.05). This consistent difference indicates that the stable, slow-moving water in the lake supports the accumulation of toxic cyanobacteria, whereas the continuous flow in rivers generally prevents these populations from establishing large biomass [25].

**Figure 2:**
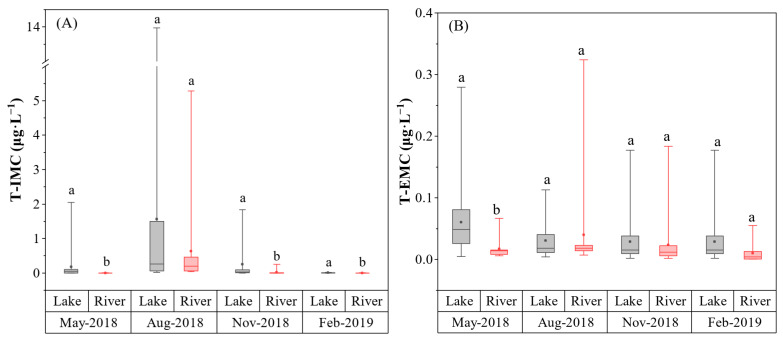
Seasonal dynamics of microcystin concentrations in Lake Taihu and its connected rivers. (**A**) Total intracellular microcystins (T−IMC). (**B**) Total extracellular microcystins (T−EMC). The box plots indicate the median (line within the box), mean (small square), 25th and 75th percentiles (box boundaries), and range (whiskers). Different lowercase letters (a, b) indicate statistically significant differences (*p* < 0.05) between lake and river sites within the same sampling month. Note the different scales on the *y*-axes.

However, this spatial pattern was disrupted in August. During this period, although the maximum T−IMC was recorded in the lake, no statistically significant difference was observed between the two zones. The river network exhibited extreme variability in T−IMC levels during August (Figure 2A), likely reflecting the hydrodynamic disturbances and biomass transport caused by the typhoon event mentioned earlier. Strong winds and heavy rainfall during the typhoon likely transported algal biomass from the lake into the outflow rivers or stirred up sediment-dwelling cyanobacteria in the tributaries, leading to similar toxin levels across the entire watershed [13,27].

**Figure 3:**
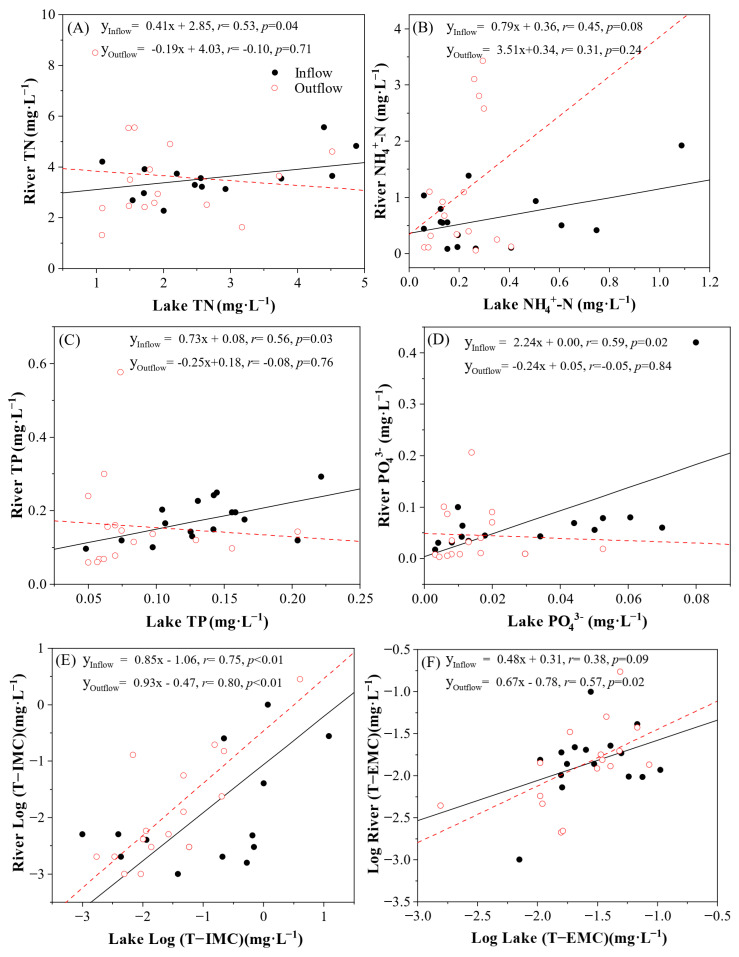
Linear regression analyses of nutrient and microcystin concentrations between Lake Taihu and its connected rivers. (**A**) Total Nitrogen (TN), (**B**) Ammonium (NH_4_^+^-N), (**C**) Total Phosphorus (TP), (**D**) Phosphate (PO_4_^3−^), (**E**) Total Intracellular Microcystins (T−IMC, log-transformed), and (**F**) Total Extracellular Microcystins (T−EMC, log-transformed). Black dots represent inflow rivers, and red circles represent outflow rivers. Black solid lines and red dashed lines represent the linear regression fits for inflow and outflow rivers, respectively.

Regarding the dissolved fraction, T−EMC concentrations generally paralleled the intracellular patterns but at a much lower magnitude (Figure 2B). A significant spatial difference was observed only during the early bloom phase in May, where T−EMC in the lake significantly exceeded that in the rivers (*p* < 0.05). In the subsequent seasons (August, November, and February), T−EMC levels were comparable between the lake and river networks (Figure 3B), suggesting that dissolved toxins were either rapidly diluted in the lotic environment or released at low levels uniformly across the watershed. This aligns with the findings of Wan et al. [32], who noted that the dilution effect driven by hydrologic transport often results in significantly lower concentrations of MCs in water bodies connected to the Yangtze River, compared to those with restricted hydrologic connectivity.

### 2.4. Spatial Heterogeneity and Coupling of Nutrients and Microcystins in the Lake–River Network

A comprehensive spatial comparison revealed a distinct functional divergence between the lake zones and their connected river networks, characterized by a “nutrient source–biological sink” pattern. As shown in Table 2, annual mean concentrations of nutrients, including TN, TP, and dissolved forms, were generally higher in the inflowing rivers compared to their corresponding lake zones, identifying the rivers as the primary external nutrient loaders. For instance, in Xukou Bay, the riverine TN concentration (3.24 ± 2.30 mg·L^−1^) was more than double that of the lake zone (1.53 ± 0.46 mg·L^−1^), demonstrating a sharp gradient of nutrient input.

In contrast, biological indicators exhibited the opposite trend. Chl-*a* and MC concentrations (both IMC and EMC) were consistently higher in the lake zones than in the rivers. Zhushan Bay, a hyper-eutrophic zone, exemplified this contrast, with mean lake IMC levels reaching 3.04 ± 5.69 mg·L^−1^ compared to only 0.07 ± 0.17 mg·L^−1^ in the connected rivers. This spatial heterogeneity confirms that while rivers supply the chemical substrate for eutrophication, the lake might act as the primary reactor for cyanobacterial proliferation and toxin production. This decoupling is consistent with the “Residence Time Hypothesis.” This hypothesis suggests that although nutrient availability is high, the continuous flow in rivers flushes out phytoplankton biomass before it can accumulate. In contrast, the static environment of the lake provides the necessary physical stability for *Microcystis* to utilize its buoyancy and form dense blooms [25].

To further elucidate the connectivity between these ecosystems, linear regression analyses were conducted separately for inflow and outflow rivers (Figure 3). Nutrient concentrations (TN, TP, and PO_4_^3−^) in inflow rivers showed significant positive correlations with those in the adjacent lake zones (*p* < 0.05), whereas no such correlation was found for outflow rivers. This statistically confirms that nutrient dynamics in the lake are largely driven by external riverine inputs. Specifically, riverine inputs of TP (r = 0.56, *p* = 0.03) and PO_4_^3−^ (r = 0.59, *p* = 0.02) were identified as key predictors of limnetic nutrient status. These findings corroborate long-term observations in the Taihu basin. They indicate that reducing external riverine loading, particularly phosphorus from agricultural and domestic sources, remains the prerequisite for mitigating lake eutrophication as the internal cycling of the lake is continually fueled by these tributary inputs [21,22,32].

Conversely, the coupling mechanism for MCs differed significantly from that of nutrients. Total intracellular MCs (T−IMC) in the river networks exhibited a strong, positive log-linear relationship with lake concentrations for both inflow (r = 0.75, *p* < 0.01) and outflow (r = 0.80, *p* < 0.01) rivers. This strong bidirectional coupling suggests that the lake determines the toxin burden of the entire watershed. For outflow rivers, the correlation reflects the direct export of toxic cyanobacteria from the lake. For inflow rivers, the high correlation (r = 0.75) implies that despite the net flow direction towards the lake, hydrodynamic exchanges or regional-scale blooms can significantly influence the water quality in the lower reaches of inflowing tributaries. This phenomenon is likely driven by wind-induced seiches or backflow events during periods of low river discharge. These hydrodynamic processes can push toxin-laden lake water upstream into the river mouths, indicating that the river-lake interface is a dynamic continuum where pollutants can spill over in both directions rather than a rigid one-way boundary [15].

In contrast, total extracellular MCs (T−EMC) showed a weaker spatial linkage, with significant correlations observed only in outflow channels (r = 0.57, *p* = 0.02), likely due to rapid dilution or degradation of dissolved toxins in the lotic environment [33]. This observation aligns with previous studies suggesting that dissolved MCs are more susceptible to biodegradation. The distinct microbial biofilms present in river sediments degrade dissolved toxins more efficiently than those in the water column, which limits their long-distance transport relative to cell-bound toxins [34].

### 2.5. Drivers of Harmful Cyanobacteria and Microcystins in the Lake–River Network

**Figure 4:**
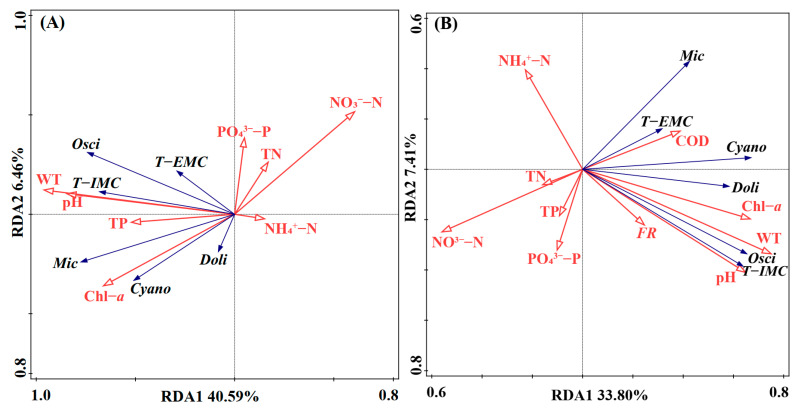
Redundancy Analysis (RDA) biplots illustrating the relationships between potentially toxigenic cyanobacteria, microcystins, and environmental variables. (**A**) Lake Taihu zones. (**B**) Connected river networks. Red arrows represent environmental factors, and blue arrows represent biological variables. Abbreviations: *Mic* (*Microcystis*); *Osci* (*Oscillatoria*); *Doli* (*Dolichospermum*); *Cyano* (Total Cyanobacteria); T−IMC (Total Intracellular Microcystins); T−EMC (Total Extracellular Microcystins); FR (Flow Rate).

Redundancy Analysis (RDA) was employed to elucidate the complex relationships between environmental drivers and biological variables in the lentic (lake) and lotic (river) ecosystems. In the lake zone (Figure 4A), the first two canonical axes explained 47.05% of the total variance in the biological data. The vectors for *Microcystis*, total cyanobacteria, Chl-*a*, and T−IMC pointed in the same direction as WT and pH, indicating that water temperature (WT) and pH emerged as the most significant positive drivers for cyanobacterial growth and toxin production. The significant positive correlation of WT and pH with cyanobacterial growth and toxin production is consistent with previous findings in Lake Taihu [28] and other hyper-eutrophic shallow lakes like Lake Erie [29], where elevated temperatures and pH-driven carbon feedback loops reinforce *Microcystis* dominance. Physiologically, this observation is consistent with physiological studies demonstrating that *Microcystis* possesses efficient carbon concentrating mechanisms (CCMs) [3,4]. These mechanisms allow it to thrive under high pH conditions where free CO_2_ is limited and give it a competitive edge over other phytoplankton during dense summer blooms [35].

Conversely, nutrient concentrations (TN, NO_3_^−^-N, NH_4_^+^-N, and dissolved phosphorus) exhibited strong negative correlations with biological parameters. This phenomenon aligns with the recent findings of Deng et al. [36], who highlighted that such nutrient depletion, acting in synergy with climate warming, is a critical driver altering the spatiotemporal dynamics of MCs in Lake Taihu. Alternatively, it reflects a seasonal mismatch where nutrient loading peaks in winter due to lower biological retention while algal growth peaks in summer [21]. This pattern highlights that in hyper-eutrophic systems like Lake Taihu, internal recycling and rapid biological assimilation can mask the positive relationship between external nutrient loading and algal biomass during the growing season [20,23].

In the connected river networks (Figure 4B), the environmental driving mechanism showed both similarities and distinct differences compared to the lake. Similarly to the lake, WT and pH remained strong positive drivers for biomass and toxins. However, Flow Rate emerged as a critical river-specific factor. The RDA vector for flow rate showed a strong positive correlation with *Oscillatoria*, *Dolichospermum*, and T−IMC, but a weaker relationship with *Microcystis*. This finding is significant as it provides statistical evidence for the hydrodynamic hypothesis proposed in Section 2.2: unlike *Microcystis* colonies, which prefer static water, filamentous *Oscillatoria* in these rivers are likely benthic or epiphytic species that are resuspended or transported into the water column under high-flow conditions (e.g., during typhoon events). Ecologically, this distinction supports the concept of hydraulic sorting. While colonial cyanobacteria require water column stability to regulate buoyancy, filamentous forms are often adapted to higher shear stress environments or are derived from periphytic mats that detach during high-discharge events [37]. This hydrodynamic differentiation is corroborated by broader regional patterns observed across the lake groups in the middle and lower reaches of the Yangtze River. Previous investigations have demonstrated that lakes maintaining hydrologic connectivity with the Yangtze River tend to be dominated by toxic filamentous cyanobacteria, whereas semi-enclosed lakes characterized by long water residence times (Lake Taihu and Lake Chaohu) are predominantly dominated by colonial *Microcystis* [33,38].

Consequently, the elevated T−IMC levels in rivers during the wet season were co-driven by high temperatures and hydrodynamic disturbances that facilitated the transport of toxigenic filamentous cyanobacteria. Additionally, unlike the strong nutrient-depletion pattern in the lake, the nutrient vectors in the rivers showed shorter projections relative to the biological axis, implying that nutrients are relatively abundant in the river network and are less likely to be the limiting factor for cyanobacterial growth compared to physical factors like temperature and flow velocity. In such nutrient-saturated lotic systems, algal growth is typically controlled by physical constraints such as light availability (turbidity) and hydraulic retention time rather than chemical stoichiometry. This nutrient-saturated state stands in contrast to the findings of Li and Murdock [30] in the Cumberland River, where phytoplankton biomass was explicitly co-limited by N and P, and species dominance shifted strictly according to N:P ratios. This divergence highlights a critical ecological threshold: in nutrient-limited lotic systems, chemical stoichiometry regulates community structure; however, in hyper-eutrophic networks like the ones in this study, nutrient concentrations exceed biological demand, causing physical controls to override stoichiometric constraints. This suggests that nutrient reduction strategies alone may be less immediately effective in controlling riverine biomass without addressing hydrological residence times [39].

### 2.6. Spatio-Temporal Variations of Human Health Risk

Human health risks associated with potential water ingestion were evaluated using the Hazard Quotient (HQ). Consistent with body weight differences, children were identified as the most vulnerable group, exhibiting substantially higher HQ values than adults across all sampling sites. This disparity is primarily due to the lower body weight of children and their relatively higher water intake rates per unit of body mass, which amplifies the toxicological burden of equivalent exposure [6].

**Figure 5:**
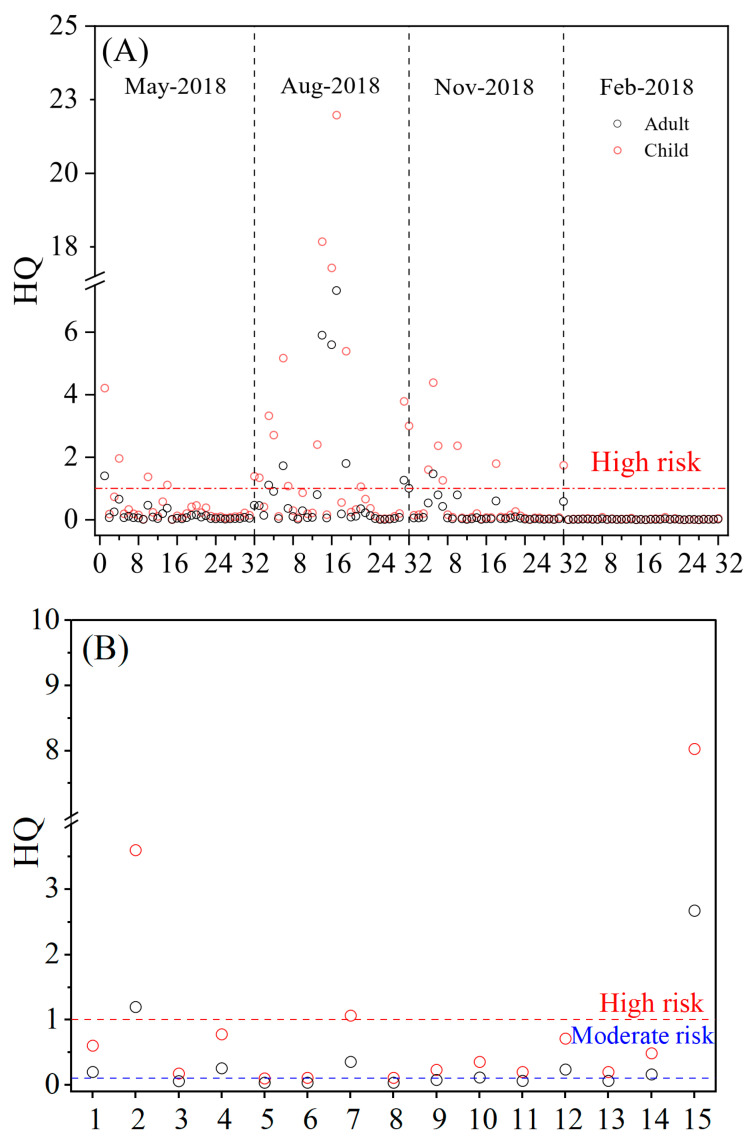
Human health risk assessment based on Hazard Quotient (HQ) for microcystins via accidental water ingestion. (**A**) Spatiotemporal variation in HQ in Lake Taihu across four seasons. (**B**) Spatial variation in HQ in connected rivers during August. The red dashed line represents the high-risk threshold (HQ = 1). Open circles represent calculated HQ values for adults (black) and children (red). Note that the river assessment was conducted specifically for August due to the high biomass observed during this period.

In the lake zone (Figure 5A), the health risk profile exhibited distinct seasonal patterns. August emerged as the most critical period, where HQ values frequently exceeded the safety threshold (HQ > 1). Specifically, the highest risk was recorded at Site 17 in Zhushan Bay, with HQ values reaching peak levels of 21.98 for children and 7.33 for adults, indicating a severe potential for adverse health effects. As a semi-enclosed bay located in the downwind direction of the prevailing summer monsoon, Zhushan Bay acts as a physical trap for drifting cyanobacterial scums, leading to the hyper-accumulation of biomass and toxins in this region [40]. High-risk sites were also prevalent in May and November, whereas February was the only month where risks were generally negligible across the lake zone.

Regarding the connected rivers, the spatial distribution of risk was highly heterogeneous (Figure 5B, assessed for August). While most riverine sites remained within safe limits due to the dilution effect of flowing water, two specific channels emerged as distinct high-risk hotspots (HQ > 1). Spatially, both of these high-risk rivers are directly connected to the northern bays of Lake Taihu (e.g., Zhushan and Gonghu Bays), with these regions most severely affected by cyanobacterial blooms. The Li River (R15), in particular, exhibited the highest risk, with children’s HQ reaching 8.03. This elevated risk is attributed to a dual mechanism: its spatial proximity to the hyper-eutrophic northern lake zones and its unique semi-lentic hydrology. With its slow currents and persistent blooms, the Li River creates a stable environment that supports continuous algal growth. This implies that such semi-closed rivers connected to eutrophic lakes act as hidden reservoirs of toxicity, posing health risks far greater than typical flowing rivers [41]. Crucially, the potential hazard extends beyond direct water exposure. Given that these riparian zones are often utilized for agriculture, the riverine transport of MCs facilitates their entry into surrounding soil and plant systems via irrigation. Previous studies have indicated that MCs can accumulate in soils and transfer to crops (bioaccumulation), thereby posing a secondary, yet significant, threat to human health through the food chain [42,43]. This suggests that the actual health burden in these hotspots may be underestimated if only aquatic exposure is considered.

## 3. Conclusions

**Figure 6:**
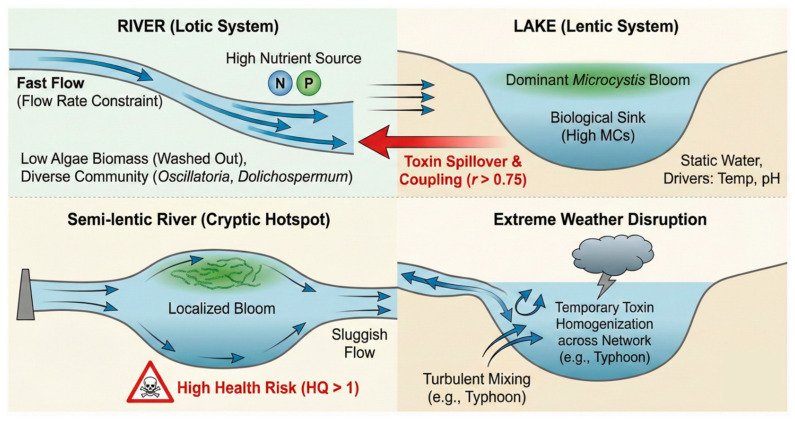
Schematic representation of source–sink dynamics and hydrodynamic regulation of harmful cyanobacteria across the Lake Taihu watershed. The model depicts (1) the distinct ecological niches between lotic and lentic systems, (2) the bidirectional coupling of toxins driven by hydrological connectivity, and (3) the impact of anthropogenic regulation and climatic disturbances on reshaping spatiotemporal risk patterns. Blue arrows illustrate hydrodynamic flow patterns and turbulence, while the red arrow and symbols denote critical toxin spillover pathways and high-risk health hazards (HQ > 1).

This study demonstrates a distinct functional divergence in the Lake Taihu watershed, where rivers act as primary nutrient sources while the lake functions as the main reservoir for cyanobacterial blooms and MCs. Despite a higher nutrient loading in rivers, their lotic nature significantly constrained algal biomass and toxin accumulation compared to the lentic lake zone, with redundancy analysis confirming flow rate as the critical limiting factor. However, distinct biological coupling was observed; IMC concentrations in connected rivers were strongly positively correlated with those in the lake, indicating that the lake exports toxicity to the surrounding watershed and vice versa in bidirectional rivers. Crucially, this spatial heterogeneity was disrupted by specific hydrological anomalies: the semi-lentic Li River exhibited high toxin levels and health risks comparable to hyper-eutrophic lake bays due to its sluggish flow, while extreme weather events temporarily homogenized toxin distribution across the network. The complex source–sink dynamics, bidirectional toxin coupling, and hydrodynamic regulation mechanisms across the lake–river continuum are visually synthesized in a schematic model (Figure 6). These findings underscore that while nutrients fuel the system, hydrodynamic conditions and connectivity ultimately determine the spatiotemporal distribution of health risks in the lake–river interface. Therefore, management strategies should shift towards an integrated watershed approach, prioritizing hydraulic flushing to disrupt riverine blooms, extending monitoring to cryptic semi-lentic hotspots, and controlling the lake–river interface to mitigate toxin spillover.

## 4. Materials and Methods

### 4.1. Study Area and Sampling Sites

**Figure 7:**
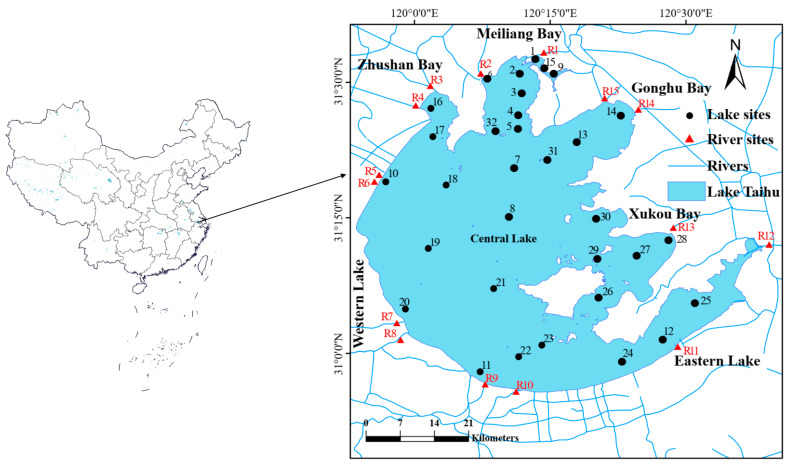
Geographical location of the study area and distribution of sampling sites in the Lake Taihu watershed. Black circles represent lake sampling sites (numbered 1–32), while red triangles denote riverine sampling sites (labeled R1–R15).

The sampling network was aligned with the long-term monitoring stations of the Taihu Laboratory for Lake Ecosystem Research (TLLER), Chinese Academy of Sciences (Figure 7). Reflecting the spatial heterogeneity of the ecosystem, the lake was partitioned into seven ecologically and limnologically distinct zones, following the established zoning framework of Qin et al. [40]. To characterize the land–water exchange, 15 major inflow and outflow rivers were selected based on their high discharge volumes, strong hydraulic connectivity to eutrophic bays, and significant anthropogenic influence [35]. Sampling at these river sites was performed synchronously with the lake stations. River sampling stations were positioned approximately 1–5 km from the lake–river interface to minimize the immediate mixing influence of lake water (e.g., backflow driven by wind) while accurately representing riverine inputs/outputs [44]. Detailed coordinates and classifications of all sampling sites are provided in Table S1.

### 4.2. Sample Collection

Seasonal sampling campaigns were conducted to capture a complete annual cycle: May 2018 (spring), August 2018 (summer), November 2018 (autumn), and February 2019 (winter). At each site, surface water samples (0–0.5 m depth) were collected using a clean Plexiglass sampler. Approximately 5 L of water per site was immediately transferred into acid-washed, amber polyethylene bottles and transported to the laboratory at 4 °C for subsequent analysis.

### 4.3. Determination of Environmental Parameters

Environmental variables were characterized through a combination of in situ measurements and laboratory analyses. Physical parameters, including water temperature (WT) and pH, were measured in situ using a multi-parameter water quality sonde (YSI EXO2, Yellow Springs Instruments, Yellow Springs, OH, USA) at each sampling site. Water samples for nutrient analysis were transported to the laboratory at 4 °C. Total nitrogen (TN), ammonium (NH_4_^+^-N), nitrate (NO_3_^−^-N), total phosphorus (TP), orthophosphate (PO_4_^3−^-P), chemical oxygen demand (COD), and chlorophyll-a (Chl-*a*) were determined following standard methods [45]. The flow rates for the 15 rivers were obtained from the study by Yan et al. [17].

### 4.4. Phytoplankton Identification

Phytoplankton samples (1 L) were immediately preserved with 1.5% acidic Lugol’s solution in the field. In the laboratory, samples were sedimented for 48 h and concentrated to a final volume of 30 mL. Taxonomic identification and cell enumeration were performed using a counting chamber under an optical microscope (Olympus BX53, Olympus Corporation, Tokyo, Japan) at 400× magnification. Algae were identified to the species or genus level according to standard taxonomic keys [46].

### 4.5. Extraction and Quantification of Microcystins

MCs were analyzed in two forms: intracellular MCs (IMCs) and extracellular MCs (EMCs). For IMCs, algal cells retained on GF/C filters (Whatman, Maidstone, UK) were lyophilized and extracted three times using 75% aqueous methanol with ultrasonication and freeze–thaw cycles [28]. The supernatants were pooled and diluted. For EMCs, 1 L of filtered water was directly processed. Both IMC and EMC extracts were purified and enriched using solid-phase extraction (SPE) cartridges (Oasis HLB, Waters Corporation, Milford, MA, USA). The cartridges were pre-conditioned, loaded, washed, and finally eluted with methanol. The eluates were evaporated to dryness under nitrogen gas and reconstituted in methanol.

The quantification of MC congeners (including MC-LR, MC-RR, and MC-YR) was performed using Ultra-High Performance Liquid Chromatography coupled with Tandem Mass Spectrometry (UHPLC-MS/MS, Waters Acquity UPLC-Triple Quadrupole MS, Waters Corporation, Milford, MA, USA) operating in positive electrospray ionization (ESI+) mode. Separation was achieved on a C18 column (e.g., BEH C18, 1.7 µm) with a gradient elution of acetonitrile and water containing 0.1% formic acid. The method detection limit (MDL) was 1 ng/L. Quality assurance/quality control (QA/QC) procedures included the analysis of procedural blanks, matrix spikes, and calibration standards after every 15 samples, with recovery rates ranging from 78% to 110%.

### 4.6. Human Health Risk Assessment

Health risks posed by the direct ingestion of MC-contaminated water were assessed using the Hazard Quotient (HQ) model [33,47]. Following the risk ranking framework established by Xiang et al. [39], health risks were categorized as: low (HQ < 0.1), moderate (0.1 ≤ HQ < 1), and high (HQ > 1). The specific calculation formulas and parameters used for the exposure assessment are detailed in the Supplementary Information.

### 4.7. Statistical Analysis

Data are expressed as mean ± standard deviation (SD). Prior to analysis, variables were log_10_(x + 0.001) transformed to satisfy the assumptions of normal distribution and homogeneity of variance. Paired-samples *t*-tests were performed using IBM SPSS Statistics 22.0 to evaluate the differences in environmental and biological parameters between the lake and connected river networks. To identify key environmental drivers, Redundancy Analysis (RDA) with Monte Carlo permutation tests (499 permutations) was conducted using Canoco 5.0 (Microcomputer Power, Ithaca, NY, USA).

To quantitatively assess the coupling relationship between the lotic and lentic systems, linear regression analyses were conducted. Specifically, the regression models were built using the seasonal mean values of indices from each lake zone and its corresponding connecting rivers to minimize spatial heterogeneity noise. Pearson correlation coefficients (*r*) were calculated to determine the strength of these associations. All statistical tests were two-tailed with significance defined at *p* < 0.05, and figures were generated using OriginPro 2026 (OriginLab Corp., Northampton, MA, USA).

## Figures and Tables

**Table 1:** Spatiotemporal variations in key physicochemical parameters in Lake Taihu and its connected rivers during the study period (May 2018–February 2019).

Date	Sites	WT (°C)	pH	TN (mg·L^−1^)	NH_4_^+^-N (mg·L^−1^)	NO_3_^−^ (mg·L^−1^)	TP (mg·L^−1^)	PO_4_^3−^ (mg·L^−1^)	Chl-*a* (mg·L^−1^)
May 2018	Lake	24.7 ± 0.6	9.3 ± 0.5 *	3.00 ± 2.14	0.15 ± 0.09 *	0.28 ± 0.23 *	0.14 ± 0.12	0.01 ± 0.00 *	102.8 ± 104.1 *
	River	25.0 ± 1.3	7.9 ± 0.4	3.50 ± 1.21	0.87 ± 0.77	0.78 ± 0.49	0.16 ± 0.08	0.04 ± 0.03	22.6 ± 16.8
August 2018	Lake	28.7 ± 0.8	8.6 ± 0.3	1.68 ± 0.66 *	0.18 ± 0.07	0.23 ± 0.19 *	0.13 ± 0.05 *	0.03 ± 0.03 *	30.0 ± 11.9 *
	River	29.7 ± 2.6	8.3 ± 0.4	3.31 ± 3.52	0.25 ± 0.51	0.48 ± 0.35	0.20 ± 0.24	0.07 ± 0.10	89.2 ± 192.8
November 2018	Lake	13.9 ± 0.4	7.2 ± 0.4	1.99 ± 1.28 *	0.33 ± 0.28 *	0.25 ± 0.30 *	0.12 ± 0.09 *	0.02 ± 0.02 *	46.3 ± 59.0 *
	River	14.4 ± 1.0	6.5 ± 0.3	3.35 ± 1.52	0.98 ± 0.74	0.93 ± 0.56	0.21 ± 0.22	0.09 ± 0.13	13.5 ± 14.5
February 2019	Lake	6.7 ± 0.7	7.3 ± 0.3	2.84 ± 1.08	0.24 ± 0.29	0.85 ± 0.50	0.08 ± 0.03 *	0.02 ± 0.02	11.3 ± 6.4 *
	River	7.4 ± 0.6	7.3 ± 0.2	3.45 ± 1.12	0.31 ± 0.33	1.07 ± 0.52	0.13 ± 0.07	0.02 ± 0.02	18.8 ± 14.7

Asterisks (*) indicate statistically significant differences (*p* < 0.05) between lake and river sites within the same sampling month.

**Table 2:** Comparison of annual mean concentrations of nutrient and microcystin between different lake zones and their connected river networks.

Lake Zones	Sites	TN (mg·L^−1^)	NH_4_^+^-N (mg·L^−1^)	NO_3_^−^ (mg·L^−1^)	TP (mg·L^−1^)	PO_4_^3−^ (mg·L^−1^)	Chl-*a* (mg·m^−3^)	IMC (μg·L^−1^)	EMC (μg·L^−1^)
Gonghu Bay	Lake	2.18 ± 1.13 *	0.11 ± 0.11 *	0.33 ± 0.33	0.08 ± 0.05	0.01 ± 0.01 *	55.9 ± 71.1	1.067 ± 2.553	0.046 ± 0.048
	River	3.89 ± 4.53	0.65 ± 0.69	0.36 ± 0.26	0.22 ± 0.32 *	0.06 ± 0.13	117 ± 258.7	0.701 ± 1.855	0.058 ± 0.109
Meiliang Bay	Lake	2.01 ± 0.83	0.13 ± 0.06 *	0.26 ± 0.24 *	0.11 ± 0.05	0.01 ± 0.01 *	50.68 ± 54.5	0.604 ± 0.771	0.049 ± 0.065 *
	River	3.15 ± 1.51	0.47 ± 0.31	0.82 ± 0.67	0.14 ± 0.16	0.03 ± 0.02	27.8 ± 19.0	0.249 ± 0.602	0.011 ± 0.005
Zhushan Bay	Lake	4.89 ± 2.12	0.45 ± 0.46 *	1.07 ± 0.42	0.22 ± 0.14 *	0.06 ± 0.03 *	77.1 ± 120.6	3.042 ± 5.689	0.026 ± 0.019
	River	4.45 ± 0.92	0.99 ± 0.66	1.20 ± 0.38	0.37 ± 0.23	0.16 ± 0.15	20.4 ± 19.0	0.07 ± 0.165	0.015 ± 0.011
Northwestern Lake	Lake	2.70 ± 0.94	0.42 ± 0.34	0.52 ± 0.38 *	0.13 ± 0.04 *	0.03 ± 0.02 *	40.8 ± 37.5 *	0.471 ± 0.706 *	0.037 ± 0.033
	River	3.17 ± 0.54	0.45 ± 0.32	0.89 ± 0.42	0.18 ± 0.06	0.06 ± 0.02	16.7 ± 7.5	0.012 ± 0.018	0.035 ± 0.06
Southwestern Lake	Lake	2.77 ± 2.16	0.26 ± 0.14	0.55 ± 0.60 *	0.13 ± 0.09	0.03 ± 0.03	55.9 ± 101.0	0.085 ± 0.116	0.034 ± 0.035
	River	3.55 ± 0.96	0.45 ± 0.51	1.03 ± 0.41	0.13 ± 0.05	0.03 ± 0.02	28.8 ± 32.9	0.053 ± 0.121	0.026 ± 0.016
Eastern Lake	Lake	1.99 ± 0.79	0.20 ± 0.15 *	0.28 ± 0.13	0.11 ± 0.10	0.01 ± 0.00	5.24 ± 4.03 *	0.044 ± 0.08	0.027 ± 0.021 *
	River	1.95 ± 0.93	0.30 ± 0.31	0.42 ± 0.46	0.07 ± 0.01	0.01 ± 0.00	4.57 ± 1.15	0.082 ± 0.126	0.011 ± 0.008
Xukou Bay	Lake	1.53 ± 0.46 *	0.30 ± 0.06 *	0.18 ± 0.09 *	0.06 ± 0.03 *	0.01 ± 0.00 *	24.8 ± 24.0	0.015 ± 0.027	0.019 ± 0.022
	River	3.24 ± 2.30	1.53 ± 1.50	0.49 ± 0.48	0.12 ± 0.08	0.05 ± 0.04	11.1 ± 11.2	0.019 ± 0.024	0.016 ± 0.014

Asterisks (*) indicate statistically significant differences (*p* < 0.05) between the lake zone and its corresponding rivers.

## Data Availability

The original contributions presented in this study are included in the article/Supplementary Materials. Further inquiries can be directed to the corresponding author(s).

## Supplementary Materials

The following supporting information can be downloaded at: https://www.mdpi.com/article/10.3390/toxins18020089/s1. Text S1: Details of Human Health Risk Assessment [33,43,47,48,49]. Table S1: Detailed geographical coordinates and characteristics of the sampling sites in the Lake Taihu watershed [17].
