# Supplementary material for: Functional Divergence and Toxin Coupling of Cyanobacterial Blooms Across the Lake–River Continuum: Insights from the Lake Taihu Watershed
Source: Toxins (Basel). 2026 Feb 9;18(2):89. doi: 10.3390/toxins18020089 (PMC12945171; doi:10.3390/toxins18020089)
Supplement: Supplementary file 1 [file toxins-18-00089-s001.zip › toxins-4114073-supplementary.pdf]

Supplementary Materials: Functional Divergence and Toxin Coupling of Cyanobacterial Blooms Across the Lake-River Continuum: Insights from the Lake Taihu Watershed

Xiang Wan, Yucong Li, Qingju Xue, Guoxiang Wang and Liqiang Xie

Text S1. Details of Human Health Risk Assessment

The HQs were calculated using Eq. (S1), assuming that surface water (as drinking water) is ingested directly by human [33, 47]:

$$HQ=CDI/RfD \quad (S1)$$

where CDI (μg/kg body weight) is the chronic daily intake of the microcystin-LR (MC-LR) through ingestion per unit body weight, and RfD (μg/(kg·d)) is the reference dose for the MC-LR through oral exposure only. The RfD was represented by the acceptable daily intake, but its value was currently available for only MC-LR (0.04 μg/kg/d) according to the World Health Organization [48]. It is not rational to use the RfD of MC-LR to evaluate health risk of the total MCs due to their different toxicities of three MC variants. The intraperitoneal half lethal doses (LD_{50}) were 43 μg/kg (MC-LR), 235.4 μg/kg (MC-RR) and 110.6 μg/kg (MC-YR) [49]. Therefore, the LD_{50} in mice for MC-RR and MC-YR is about 5- and 2.5-fold that for MC-LR, corresponding to 0.2 and 0.4 MC-LR equivalents, respectively [43]. Therefore, CDI for MC-LR equivalents was calculated by Eq. (S2)-(S3)

$$CDI= (C\times IR) / BW \quad (S2)$$

$$C=C_{MC\text{-}LR}+C_{MC\text{-}RR}\times 0.2+C_{MC\text{-}YR}\times 0.4 \quad (S3)$$

where C (μg/L) is the concentration of MC-LR equivalents in lakes, IR is the water ingestion rate (1 litre/day for a child, 2 litres/day for an adult), and BW is the body weight (10 kg for a child, 60 kg for an adult) [48].

Table S1. Detailed geographical coordinates and characteristics of the sampling sites in the Lake Taihu watershed.

Part A: Lake Sampling Sites (Sites 1–32)

Sites	Longitude° (E)	Latitude° (N)	Sites	Longitude° (E)	Latitude° (N)
1	120.21944	31.53968	17	120.03182	31.39761
2	120.19067	31.51317	18	120.05612	31.30810
3	120.19433	31.47633	19	120.02333	31.19055
4	120.18796	31.43609	20	119.96731	31.10789
5	120.18733	31.41117	21	120.14386	31.11651
6	120.13117	31.50383	22	120.18982	30.99104
7	120.18017	31.33833	23	120.23271	31.01261
8	120.17062	31.24816	24	120.37909	30.98091
9	120.25317	31.51300	25	120.51329	31.08941
10	119.94500	31.31450	26	120.33567	31.09903
11	120.11866	30.96367	27	120.40596	31.17683
12	120.45383	31.02167	28	120.464785	31.205651
13	120.29550	31.38650	29	120.33361	31.17114
14	120.37691	31.43511	30	120.33149	31.24482
15	120.23612	31.52310	31	120.24154	31.35340
16	120.02817	31.45001	32	120.14594	31.40680

Note: The 32 sampling sites were uniformly distributed across different ecological zones of the lake, including: Meiliang Bay (Sites 1–6, 9, 15, 32), Gonghu Bay (Sites 13, 14, 31), Zhushan Bay (Sites 16, 17), Northwest Zone (Sites 10, 18, 19), Southwest Zone (Sites 11, 20, 22, 23), East Taihu Lake (Sites 12, 24, 25), Xukou Bay (Sites 26–30), and Central Lake (Sites 7, 8, 21).

Part B: River Sampling Sites (Sites R1–R15)

Sites	Longitude° (E)	Latitude° (N)	River Name	Flow Pattern / Status [a]	Connected Lake Zone
R1	120.228	31.548	Liangxi	Bidirectional (Inflow dominated)	Meiliang Bay
R2	120.121	31.509	Zhihu	Inflow	
R3	120.026	31.491	Taige	Inflow	Zhushan Bay
R4	119.999	31.455	Yincun	Inflow	
R5	119.927	31.322	Chendong	Inflow	Northwest Zone
R6	119.923	31.314	Dapu	Inflow	
R7	119.964	31.053	Hexi	Bidirectional (Inflow dominated)	Southwest Zone
R8	119.971	31.022	Changxing	Bidirectional (Balanced)	
R9	120.127	30.94	Changdou	Bidirectional (Outflow dominated)	
R10	120.184	30.927	Daqian	Bidirectional (Outflow dominated)	
R11	120.482	31.009	Taipu	Outflow	East Taihu Lake
R12	120.65	31.198	Guajing	Outflow	
R13	120.474	31.229	Xujiang	Outflow	Xukou Bay
R14	120.409	31.447	Wangyu	Bidirectional (Outflow dominated)	Gonghu Bay
R15	120.347	31.468	Li	Outflow	

[a] The flow patterns and classification of inflow/outflow status were derived from the annual mean flow data reported by Yan et al. [17].
